# Luteolin as a potential host-directed immunotherapy adjunct to isoniazid treatment of tuberculosis

**DOI:** 10.1371/journal.ppat.1009805

**Published:** 2021-08-20

**Authors:** Dhiraj Kumar Singh, Sultan Tousif, Ashima Bhaskar, Annu Devi, Kriti Negi, Barnani Moitra, Anand Ranganathan, Ved Prakash Dwivedi, Gobardhan Das

**Affiliations:** 1 Special Centre for Molecular Medicine (SCMM), Jawaharlal Nehru University, New Delhi, India; 2 International Centre for Genetic Engineering and Biotechnology, New Delhi, India; 3 Southwest National Primate Research Center, Texas Biomedical Research Institute, San Antonio, Texas, United States of America; National Institutes of Health, UNITED STATES

## Abstract

Tuberculosis (TB) remains a major health problem throughout the world with one third of the population latently infected and ~1.74 million deaths annually. Current therapy consists of multiple antibiotics and a lengthy treatment regimen, which is associated with risk for the generation of drug-resistant *Mycobacterium tuberculosis* variants. Therefore, alternate host directed strategies that can shorten treatment length and enhance anti-TB immunity during the treatment phase are urgently needed. Here, we show that Luteolin, a plant-derived hepatoprotective immunomodulator, when administered along with isoniazid as potential host directed therapy promotes anti-TB immunity, reduces the length of TB treatment and prevents disease relapse. Luteolin also enhances long-term anti-TB immunity by promoting central memory T cell responses. Furthermore, we found that Luteolin enhances the activities of natural killer and natural killer T cells, both of which exhibit antitubercular attributes. Therefore, the addition of Luteolin to conventional antibiotic therapy may provide a means to avoid the development of drug-resistance and to improve disease outcome.

## Author summary

The current TB therapy is lengthy, expensive, and may induce severe hepatotoxicity in patients, often leading to premature withdrawal from therapy, which is associated with the risk of generating drug-resistant strains. We considered inclusion of a hepatoprotective immunomodulator, Luteolin, as a potential host directed adjunct to available therapy as a means to improve its efficacy by enhancing host protective immunity. Luteolin-Isoniazid combination therapy induces improved central memory T cell responses. The boosted immune responses permitted reduction of treatment duration, improved treatment outcome and efficiently prevented disease relapse. Luteolin treatment rendered the host resistant against reinfection and reactivation of the disease, which is a major challenge following conventional TB treatment. We conclude that Luteolin is an effective adjunctive immunomodulator for designing anti-TB immunotherapeutics that can provide superior host protection.

## Introduction

Tuberculosis (TB) has emerged as the greatest killer among infectious diseases, with one third of the global population infected, and 10.4 million cases and ~1.74 million deaths reported [1]. Current therapy of TB by Directly Observed Treatment Short-Course (DOTS) involves two months of intensive phase treatment with the antibiotics Isoniazid (INH), Rifampicin, Pyrazinamide, and Ethambutol, followed by four months of extensive phase treatment with INH and Rifampicin for drug-sensitive TB, and even longer for treatment of drug-resistant TB [2,3]. Because of the lengthy treatment regimen with multiple expensive antibiotics that exhibit significant toxicity, a sizable number of patients prematurely withdraw from treatment, which is a key risk factor for the generation of drug-resistant *Mycobacterium tuberculosis* (*M*.*tb*) strains [4–6]. Importantly, even after completion of DOTS, patients exhibit increased susceptibility to disease reactivation and re-infection [7–9]. These observations suggest that anti-tubercular antibiotics impair host protective immune responses. In this context, recent studies have shown that INH eliminates antigen-responding CD4^+^ T cells, which enhances the risk for post-treatment reactivation and re-infection of disease [10,11]. Another concerning issue is that DOTS does not include an immunomodulator to curtail some of the detrimental effects of the antibiotics on the host immune system. The WHO’s expert consultation on immunotherapeutic interventions for TB has recommended inclusion of immunotherapeutics in combating TB to improve treatment efficacy of drug-resistant TB, shorten treatment length, and enhance host immunity after treatment is completed [12].

It is now well-established that CD4^+^ T cells play a central role in host protective immunity against TB. Emerging studies with patients and animal models have shown that T helper 1 (Th1) cells, which are potent producers of IFN-γ, are the critical T cells that confer host protection [13–15], whereas T helper 2 (Th2) cells, which are potent producers of IL-4, together with regulatory T (Treg) cells, facilitate disease progression by inhibiting Th1 responses [15–17]. Paradoxically, TB patients exhibit strong Th1 responses, as revealed by their delayed-type hypersensitivity (DTH) responses, yet disease continues to progress. These IFN-γ-producing cells are CD62L^lo^CD44^hi^ effector memory T (T_EM_) cells [18,19]. In fact, active TB patients possess profoundly higher numbers of T_EM_ cells than latently infected individuals, who typically possess greater numbers of CD62L^hi^CD44^hi^ central memory T (T_CM_) cells [20,21]. Although effector T cell functions and IFN-γ are essential for host protection, exuberant effector T cell responses can be harmful. The clinical manifestations of TB exhibit two components: damage induced by the pathogen and damage induced by inflammation. Therefore, addition of steroids to antibiotic treatment regimens has been beneficial [22]. Thus, in addition to antibiotics, therapy of TB may be enhanced by immunomodulator(s) that halt the adverse effects of antibiotics on host immunity while enhancing T_CM_ cell responses.

T_CM_ cell responses play a critical role in driving secondary recall responses and hence the efficacy of TB vaccines [18,23]. Conventional antibiotic treatment renders patients susceptible to *M*.*tb* reactivation and re-infection, suggesting that host protective T cells, most notably T_CM_ cells, become impaired during treatment. Therefore, therapeutics that can restore host T_CM_ responses may be able to induce strong recall responses in the host, thereby reducing the risk for TB reactivation and re-infection. To provide the host with a continuous supply of T_EM_ cells, a large pool of T_CM_ cells is needed. The generation of T_EM_ cells increases with the severity of bacterial pathogenesis and these cells rapidly produce copious amounts of IFN-γ upon antigenic challenge. T_EM_ cells, however, are terminally differentiated effector cells with low or no proliferative capacity [24,25]. Therefore, maintenance of long-term protective memory responses is thought to rely on T_CM_ cells with high proliferative capacity [26]. Thus, increasing the pool of T_CM_ (CD4^+^CD62L^hi^CD44^hi^) cells by concomitant regulation of T_EM_ (CD4^+^CD62L^lo^CD44^hi^) cells may be an effective strategy to develop long-lasting and robust recall responses. A recent study showed that inhibition of Kv1.3 K^+^ ion channels, which are enriched in T_EM_ cells, by clofazimine enhances the pool of T_CM_ cells induced by the BCG vaccine, and these cells have the potential to continuously replace effector T cells at the site of infection, thereby improving host immunity [27]. Clofazimine, a well-known anti-leprotic drug, is a category 5 drug for TB and is currently only employed in long regimens to treat extremely drug-resistant (XDR)-TB. The effect of clofazimine on memory T cells during treatment of TB remains to be investigated.

Clofazimine has limited efficacy against TB and is associated with substantial side effects [27]. In an effort to avoid some of these limitations of this drug, we focused on another inhibitor of the Kv1.3 K^+^ ion channel, Luteolin, i.e. 3,4,5,7-tetrahydroxyflavone, has been shown to effectively inhibit the Kv1.3 K^+^ ion channel [28]. Luteolin has recently been employed as a food supplement and is considered safe for human use. It is a flavonoid found in many fruits, vegetables, and medicinal plants such as *Reseda luteola* L., *Achillea millefolium* L. and many others. Luteolin-rich herbal extracts have been used for a long time as traditional herbal remedies [29]. This treatment has been found efficacious in allergies, chronic inflammatory conditions, atherosclerosis, neoplastic disorders, diabetes and obesity [29–31]. Key physiological properties of Luteolin include spasmolytic, analgesic, antioxidant and cough-relieving effects in pulmonary diseases as well as anti-carcinogenic, anti-angiogenic, anti-allergic, antiviral, anti-obesity, vasodilatory, radioprotective and hepatoprotective activities [29,31]. Many of these conditions represent risk factors for TB. A recent study showed that inhibition of Kv1.3 K^+^ ion channels, which are enriched in T_EM_ cells, by Luteolin enhances the pool of T_CM_ cells induced by the BCG vaccine, and these cells have the potential to continuously replace effector T cells at the site of infection, thereby improving host immunity and improving vaccine efficacy [32]. Taken together, these observations suggest that Luteolin might have potent immunomodulatory effects along with selective enrichment of the T_CM_ pool that may be highly beneficial in combating TB. Therefore, we studied the effects of Luteolin on TB therapy, either alone or in combination with conventional antibiotics.

Our results demonstrate that Luteolin is a potent immunomodulator that assists in the clearance of *M*.*tb* from infected animals when combined with conventional antibiotics. Analysis of effector cells revealed that Luteolin promotes the activity of natural killer (NK) and natural killer T (NKT) cells, which are known to participate in host protective immune responses against TB well before the onset of adaptive T cell responses [33]. In this manuscript we have shown that adjunct therapy with Luteolin significantly enhances therapeutic efficacy of isoniazid and, most importantly, reduces the probability of disease re-activation. Concomitantly, there is an increase in T cell responses, especially central memory T (T_CM_) cells. It is well established that T_CM_ cells play important roles in host protection against TB pathogenesis [23,33–35]. Therefore, it is likely that Luteolin mediated *M*.*tb* clearance, at least in part, is achieved by T_CM_ cells. Nevertheless, blockade of Kv1.3 ion channels on effector memory T (T_EM_) cells facilitates the generation of T_CM_ cells [36,37]. Thus, blocking Kv1.3^+^ ion channels by Luteolin [28] may facilitate bacterial clearance. Treatment with Luteolin dramatically reduced susceptibility to re-infection and re-activation of TB disease as compared with animals that received conventional antibiotics only. Furthermore, animals receiving Luteolin produced significantly higher numbers of CD62L^hi^CD44^hi^ T_CM_ cells. Taken together, our findings reveal that Luteolin-based immunotherapy can enhance the efficacy of TB antibiotic treatment by inducing strong cell-mediated immunity and therefore holds promising potential for translation to human patients.

## Results

### Luteolin treatment reduces the bacterial burden in *M*.*tb* infected mice

**Fig 1:**
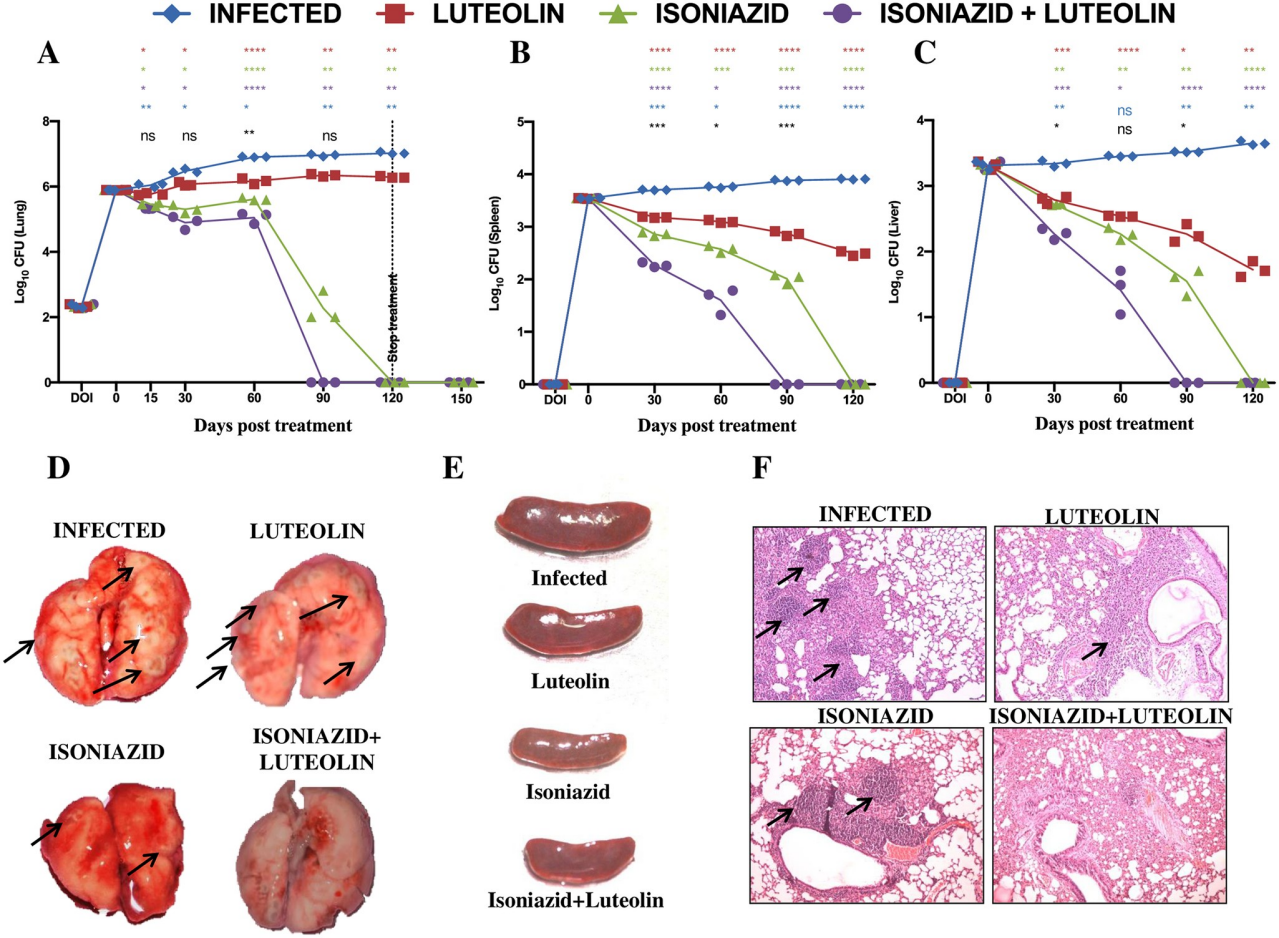
Luteolin protects mice against tuberculosis. C57BL/6 mice were challenged with H37Rv via the aerosol route with a dose inoculum of ~ 220 CFU/mice, sacrificed at various time points and lungs and spleens were harvested. Log10 CFU from the lung **(A)**, spleen **(B)** and liver **(C)** homogenates of mice infected with H37Rv and treated with Luteolin, Isoniazid (INH) or Luteolin+INH. Data shown are representative of three independent experiments with three mice per group. Differences were considered significant at p<0.05 and are represented as following: * for Infected vs. Luteolin, * for Infected vs. INH, * for Infected vs. Luteolin+INH, * for Luteolin vs. Luteolin+INH & * for INH vs. Luteolin+INH; * p<0.05, ** p<0.01, *** p<0.001, **** p<0.0001. Gross pathology of lungs **(D)** and spleen **(E)** at 60 dpi. Granuloma-like structures are indicated by arrows. **(F)** Lungs were dissected out and preserved in 4% paraformaldehyde. These preserved lungs were then processed for paraffin embedding, sectioning and staining with Hematoxylin and Eosin (H&E). Granuloma-like structures are indicated by arrows.

The available literature indicates that Luteolin is a strong immunomodulator and exhibits efficacy against disease indications that are correlated with TB susceptibility [29–31]. A recent study showed that Luteolin enhances the T_CM_ response induced by the BCG vaccine thereby improving host immunity and improving vaccine efficacy [32] while another independent report attributed enhanced NK cell and cytolytic effects to luteolin [38]. We evaluated whether Luteolin, either alone or in combination with existing antibiotics, exerts beneficial effects against primary *M*.*tb* infection. Luteolin did not exerted any bactericidal effects on *M*.*tb in vitro* in a cell free system at up to 75 μM/mL, but a significant reduction in growth was seen at a higher concentration of 100 μM/mL (S1 Fig). However, this effect did not appear to be synergistic with isoniazid (S1 Fig). We then treated mice with Luteolin at 5 mg/kg body weight using the intraperitoneal route of administration to maintain homogeneity in dosage and to achieve higher bioavailability than the oral route [29,39], starting at 15 days after infection with an aerosol dose (~220 CFU/mouse) of *M*.*tb*. Luteolin caused a transient reduction of the bacterial burden in the lungs during the initial phase of infection, which stabilized at 0.6–0.73 log colony-forming units (CFU) difference compared with vehicle-treated mice during the later phase of infection (Fig 1A–1C). However, a combination of Luteolin and INH profoundly inhibited *M*.*tb* growth and significantly reduced the time needed to clear the bacterial burden in mice (Fig 1A–1C). Effects of Luteolin, either alone or in combination with INH, in spleen and liver were even more pronounced (Fig 1B and 1C). Analysis of the gross pathology of the lungs at 60 dpi revealed fewer and smaller granuloma-like consolidations in mice treated with a combination of Luteolin and INH (Fig 1D), compared with animals that received vehicle or INH only. Splenomegaly also corresponded well with the observed bacterial burden (Fig 1E). Next, we performed histological analyses of the lungs. Mice treated with Luteolin or with Luteolin+INH showed increased numbers of infiltrating cells and reduced levels of necrosis compared with controls (Fig 1F). When analyzed at high magnification, the major fraction of infiltrating cells appeared to be of lymphoid origin. Controls showed a mixture of infiltrating cells composed mainly of macrophages and PMN leukocytes in addition to lymphocytes.

### Immunological changes following Luteolin treatment

**Fig 2:**
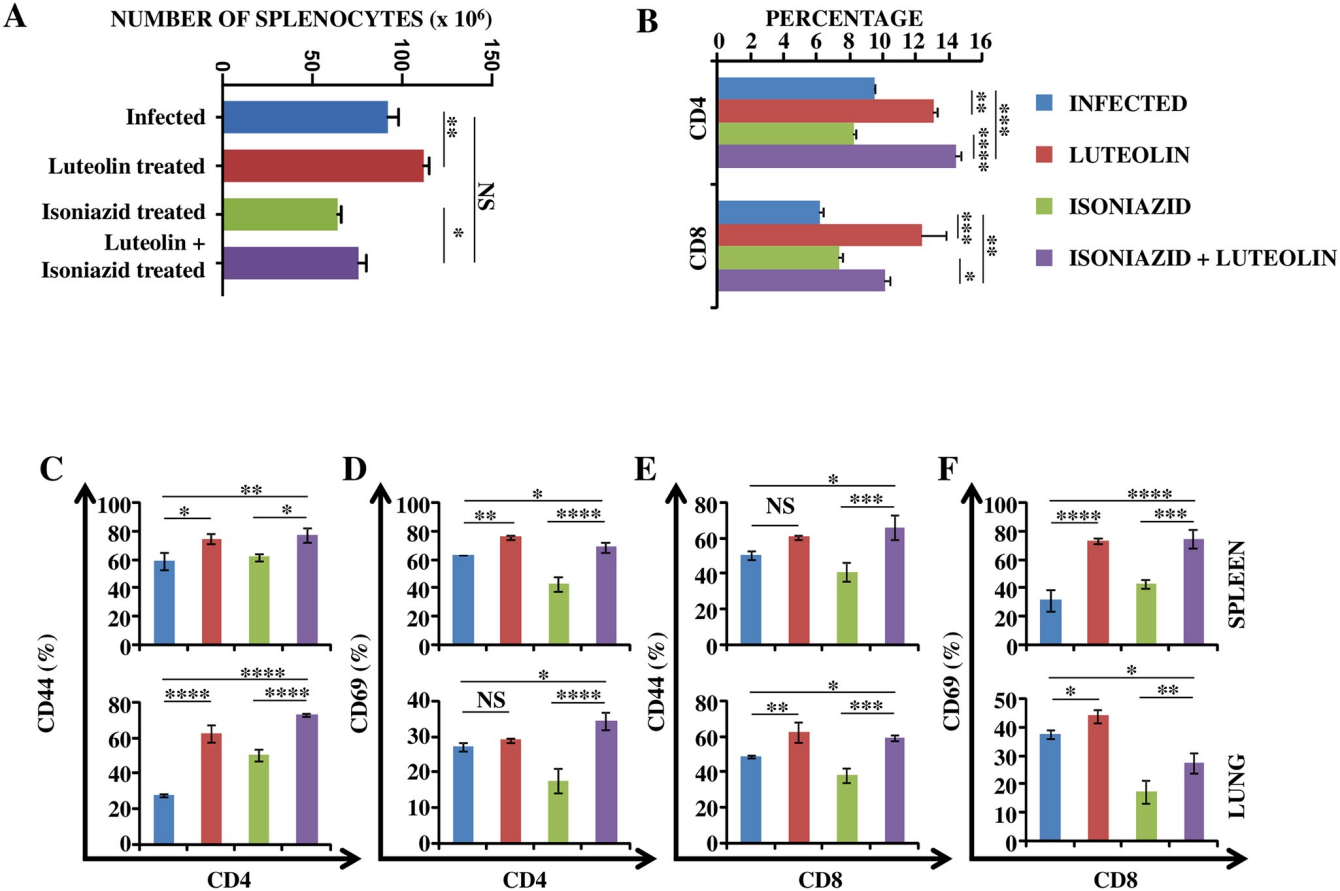
Luteolin induces protective T cell responses. T lymphocytes isolated from lungs and spleens of the indicated groups of experimental mice at 60 days post-infection were surface-stained with anti-CD4, -CD44 and -CD69 antibodies on ice and fixed prior to acquisition by flow cytometry. **(A)** Number of splenocytes in Infected, Luteolin-treated, Isoniazid-treated and Luteolin+Isoniazid-treated mice. **(B)** Percentage of CD4^+^ and CD8^+^ T cells in splenocytes of Infected, Luteolin-treated, Isoniazid-treated and Luteolin+ Isoniazid-treated mice. CD4^+^ T cell activation shown by CD44 **(C)** and CD69 **(D)** in spleen (upper panel) and lung (lower panel) of mice infected with H37Rv and treated with Luteolin, INH or Luteolin+INH. CD8^+^ T cell activation shown by CD44 **(E)** and CD69 **(F)** in spleen (upper panel) and lung (lower panel) of mice infected with H37Rv and treated with Luteolin, INH or Luteolin+INH. Data shown are representative of three independent experiments with three mice in each group and represent the MEAN±STDEV values. Differences were considered significant at P<0.05 and are represented by * p<0.05, ** p<0.01, *** p<0.001, **** p<0.0001 whereas non-significant differences are denoted by (NS).

From the preceding section it is evident that Luteolin facilitated the clearance of *M*.*tb* by INH via immunomodulation. Therefore, to better understand the immunomodulatory effect of Luteolin we assessed T cell responses *ex vivo*. We found that animals treated with Luteolin or Luteolin+INH exhibited increased numbers of total splenocytes as well as CD4^+^ and CD8^+^ T cell populations (Fig 2A and 2B and S2 Fig). Phenotypic analyses showed that the majority of T cells were activated, as revealed by surface expression levels of CD44 and CD69 in spleen (Fig 2C–2F, **upper panel**) and lungs (Fig 2C–2F, **lower panel**).

**Fig 3:**
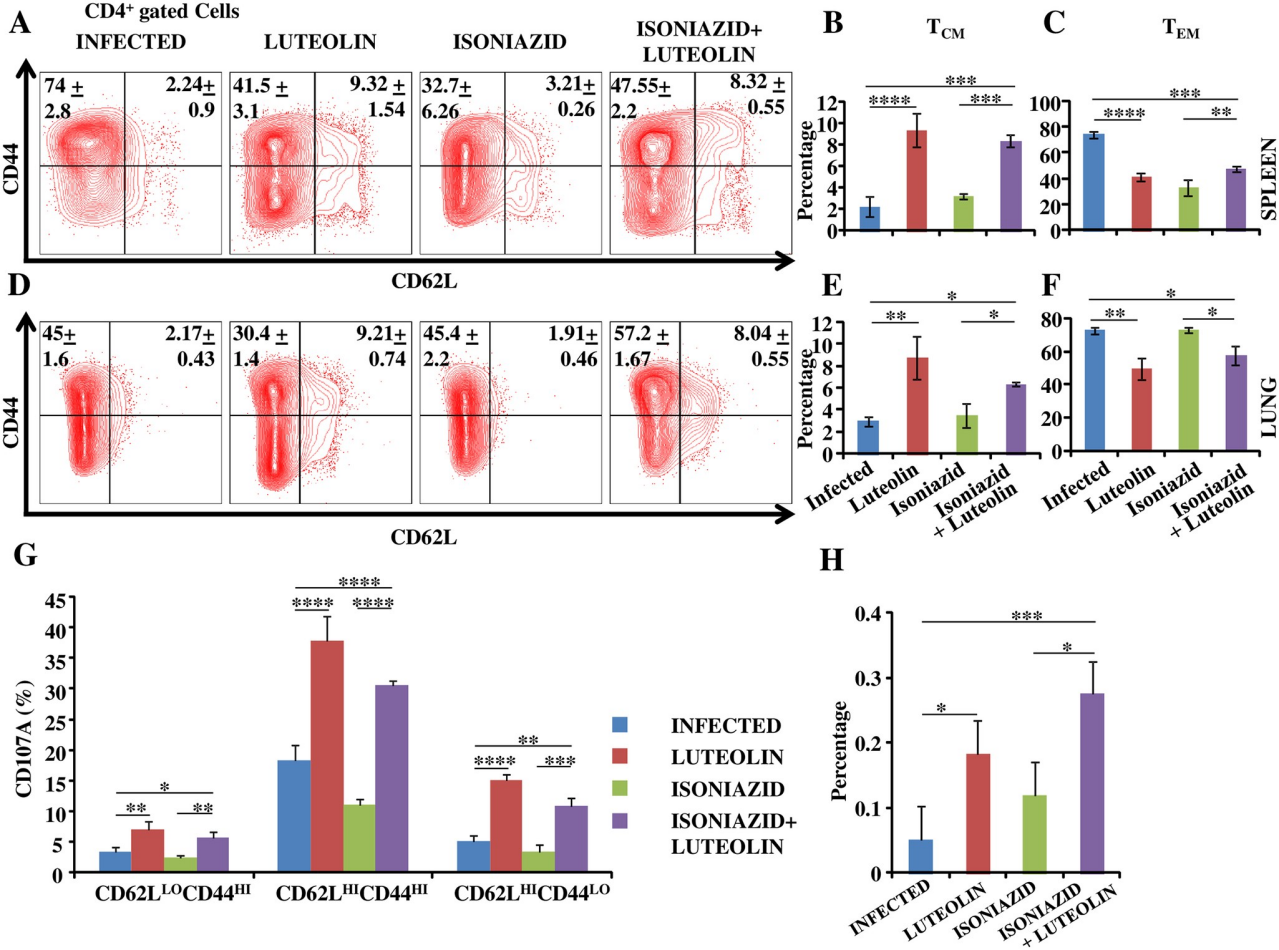
Luteolin induces superior antigen-specific memory T cell responses. T lymphocytes isolated from lungs and spleens of the indicated groups of experimental mice at 60 days post-infection were surface-stained with anti-CD3, -CD4, -CD44, -CCR7 and -CD62L antibodies on ice and fixed prior to acquisition by flow cytometry. Representative FACS profile **(A)** and proportion of central memory **(B)** and effector memory **(C)** subsets of CD4^+^ T cells in spleen. Representative FACS profile **(D)** and proportion of central memory **(E)** and effector memory **(F)** subsets of CD4^+^ T cells in lung. **(G)** *Ex-vivo M*.*tb* CSA antigen-specific degranulation in different CD4^+^ T cell subsets in lung. **(H)** Multifunctional CD4^+^ T cell populations in lung of infected, Luteolin-treated, Isoniazid-treated and Luteolin+Isoniazid-treated mice. Data shown here are representative of three independent experiments with three mice in each group and represent the MEAN±STDEV values. Differences were considered significant at P<0.05 and are represented by * p<0.05, ** p<0.01, *** p<0.001, **** p<0.0001 whereas non-significant differences are denoted by (NS).

It has recently been shown that T_CM_ cells play a critical role in host protection against TB [18,23]. We further found that both Luteolin and Luteolin+INH treated animals harboured higher proportions of CD4^+^CD62^hi^CD44^hi^ T_CM_ cells in spleen (Fig 3A and 3B) and lungs (Fig 3D and 3E) as compared with their respective controls, whereas the CD4^+^CD62^Lo^CD44^hi^ T_EM_ cell percentages were lower in both spleen (Fig 3A and 3C) and lungs (Fig 3D and 3F) of luteolin only group and lungs of Luteolin+INH group (Fig 3D and 3F). Furthermore, Luteolin treatment enhanced antigen-specific degranulation potential among these subsets in lungs (Fig 3G). Therefore, these observations further suggested that Luteolin improves host protection during therapy by promoting T_CM_ responses. Moreover, Luteolin-treated animals demonstrated more robust antigen-specific multifunctional T helper cell responses in lungs compared to controls (Fig 3H). Multifunctionality in T cells correlates strongly with a protective immune response [40].

### Luteolin expands and activates NK and NKT cells

**Fig 4:**
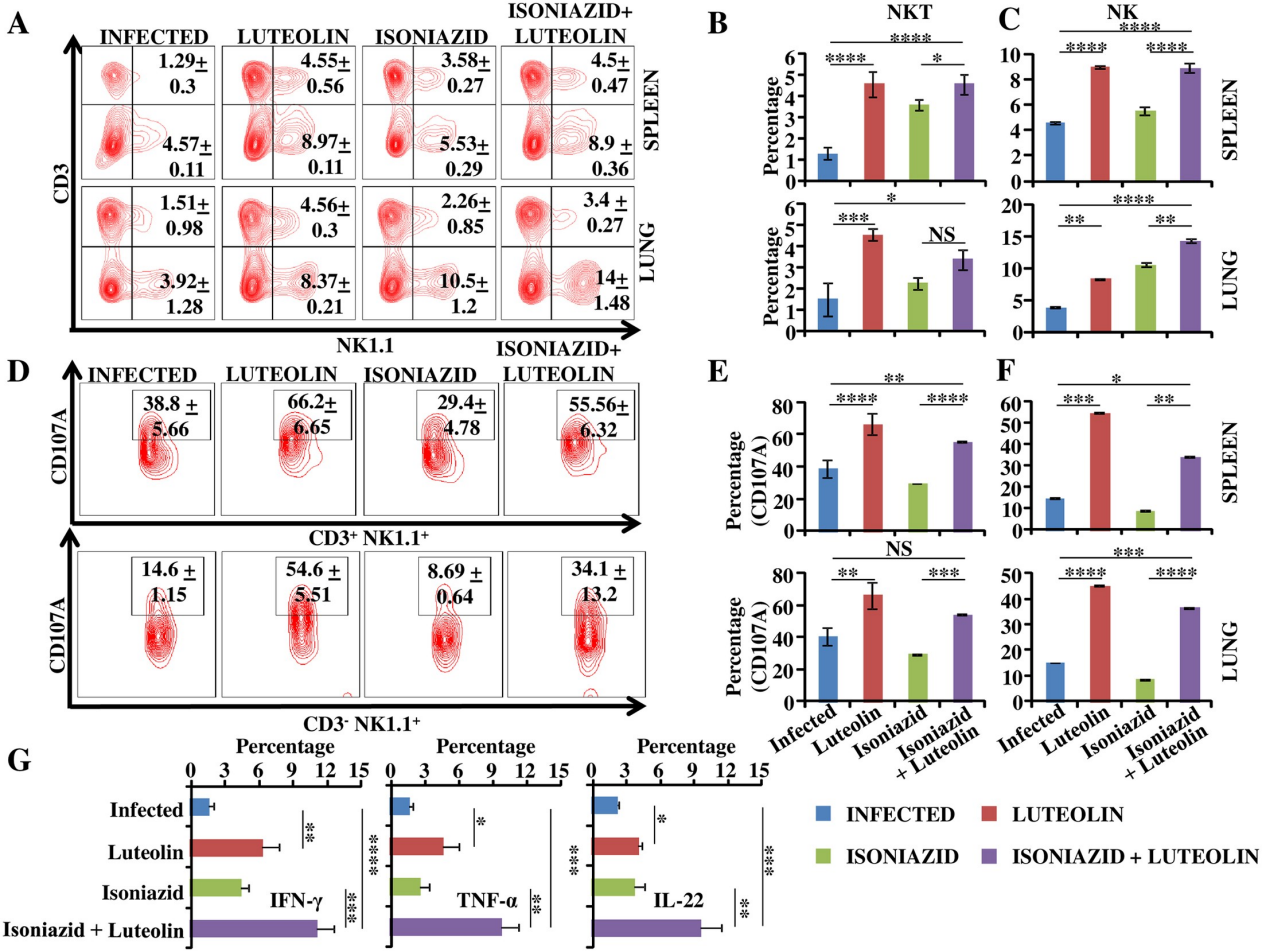
Luteolin-mediated anti-TB responses involve natural killer and natural killer T cells. Alveolar cells and splenocytes isolated from the indicated mice at 60 days post-infection were surface-stained with anti-CD3 and -NK1.1 antibodies. Representative FACS profile **(A)** and proportion of natural killer T (NKT) cells **(B)** and natural killer (NK) cells **(C)** *in vivo* in spleen (B&C, upper panel) and lung (B&C, lower panel) of different experimental groups. **(D)** Representative flow plots depicting antigen-specific degranulation potential of NKT (upper panel) and NK cells (lower panel) *ex vivo*. Fraction of degranulating NKT cells **(E)** and NK cells **(F)** in spleen (upper panel) and lung (lower panel). **(G)** Intracellular cytokine profiling of NKT cells in lungs. Data shown are representative of three independent experiments with three mice in each group and represent the MEAN±STDEV values. Differences were considered significant at P<0.05 and are represented by * p<0.05, ** p<0.01, *** p<0.001, **** p<0.0001 whereas non-significant differences are denoted by (NS).

Several recent reports have indicated that NK and NKT cells assist in host resistance against TB infection [41–46]. NK cells can specifically lyse infected cells without prior exposure, thereby playing a central role in innate immunity against TB infection [43,45–47]. NK cells may also reduce Treg expansion by direct lysis of *M*.*tb*-expanded Tregs [43,45–47], thereby shifting the initial T cell response towards the Th1 phenotype. NK cells are also capable of priming CD8^+^ T cell effector functions against *M*.*tb* [44]. While most of these functions are IL-22-mediated, these innate lymphoid cells (ILCs) alone are not considered sufficient for complete protection against TB infection. A recent report has attributed enhanced NK cell-mediated functionality to Luteolin [38]. To obtain insight in the potential effects of Luteolin on NK and NKT cells during TB, we stained lung and spleen cells with anti-CD3 and -NK1.1 antibodies. We found significantly increased numbers of both CD3^-^NK1.1^+^ and CD3^+^NK1.1^+^ cells in the spleen and lung of Luteolin-treated animals (Fig 4A–4C and S2 Fig). To further analyze these cytotoxic effector cell subsets we stained them with antibodies against the activation and degranulation marker CD107A. As expected, a large number of cells expressed CD107A and this number was significantly higher in animals that received Luteolin (Fig 4D–4F). In addition to cytotoxic effector functions, NKT cells produce copious amounts of T cell-polarizing cytokines, including both IFN-γ and IL-4, which are associated with TB host resistance and disease progression, respectively. Therefore, we examined if Luteolin induces IFN-γ and/or IL-4. Interestingly, we found that Luteolin selectively induces IFN-γ production. We also detected significantly higher levels of secreted TNF-α, a cytokine that similarly plays a protective role against TB, by NKT cells (Fig 4G).

**Fig 5:**
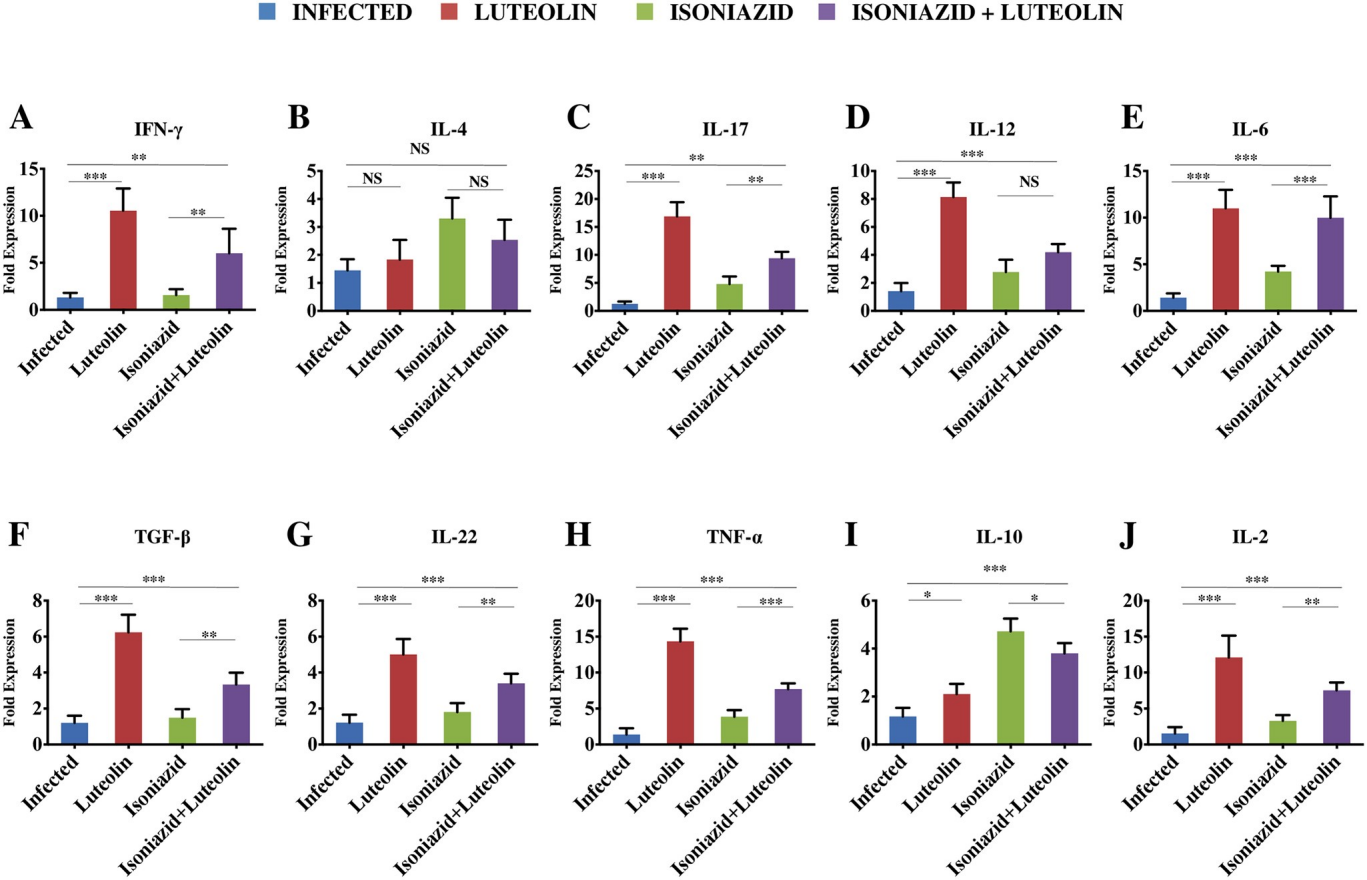
Expression of pro- and anti-inflammatory cytokines in the lungs of infected animals. The lungs from infected and treated mice were macerated and ex-vivo stimulated with *M*.*tb* complete soluble antigen for 24 h followed by RNA extraction and RT-PCR analysis (see Materials and methods). Bar graphs represent the fold change in the expression of **(A**) IFN-γ, **(B)** IL-4, **(C)** IL-17, **(D)** IL-12, **(E)** IL-6, **(F)** TGF-β, **(G)** IL-22, **(H)** TNF-α, **(I)** IL-10 and **(J)** IL-2 in various groups of mice as compared to the control infected mice. Data represents 5 mice per group (Mean ± SD, n = 5). *p<0.05, **p< 0.005, ***p<0.0005.

We also analyzed the expression of key cytokines in lungs by qRT-PCR and found that Luteolin selectively enhances the levels of Th1 (IFN-γ and TNF-α) and Th17 (IL-17 and IL-22) cells, but has only modest effects on IL-4 production (Fig 5).

### Luteolin protects antibiotic-treated animals against disease recurrence

**Fig 6:**
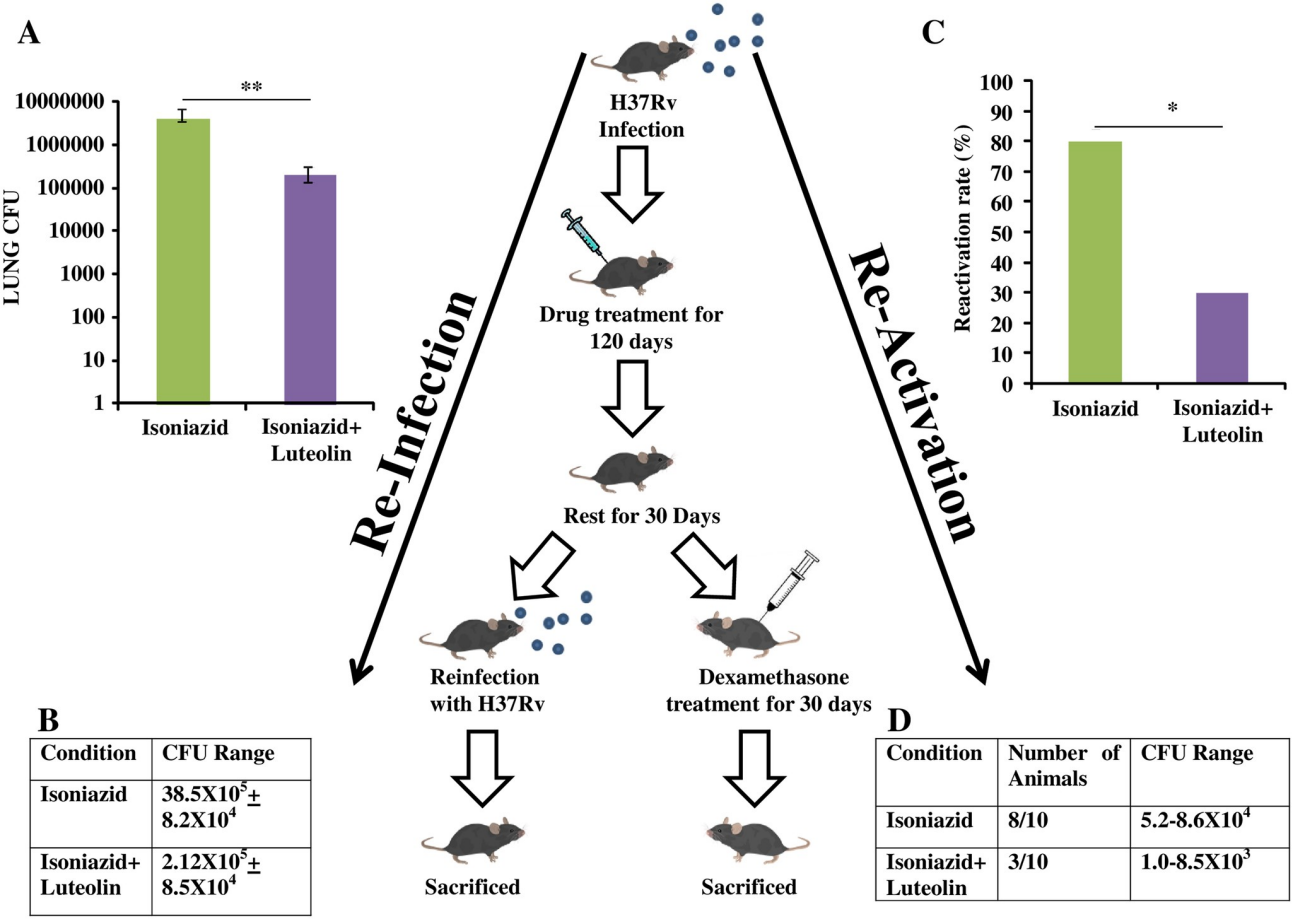
Luteolin-mediated TB immunity prevents disease relapses. *M*.*tb* infected mice were treated with Isoniazid or Isoniazid+Luteolin for 120 days and then rested for another 30 days. Then, one group was reinfected with *M*.*tb* and a second group was treated with dexamethasone for 30 days. Mice were then sacrificed for CFU estimation to determine the rate of relapse post-treatment. **(A & B)** Lung CFU in reinfected mice after 15 days of secondary infection. **(C & D)** Rate of reactivation of latent bacteria and corresponding CFU range recorded. Data shown are from a single experiment of ten mice in each group and represent the MEAN±STDEV values. Differences were considered significant at P<0.05 and are represented by * p<0.05, ** p<0.01, *** p<0.001, **** p<0.0001 whereas non-significant differences are denoted by (NS).

An effective immunomodulator for TB treatment should not only shorten treatment time for drug-sensitive TB but should also improve host immunity to protect against recurrent disease due to disease relapse (i.e., reactivation of latent bacteria) or secondary infection (i.e., reinfection) [12]. Furthermore, a stronger T_CM_ pool can efficiently drive a faster and more robust recall response which may prevent disease relapse. To investigate the extent of long term protection induced by Luteolin in preventing disease relapse we employed models of disease re-activation and reinfection. In the reinfection model, the INH-treated group established active infection with a bacterial load of 38.5X10^5^ (6.6 Log) CFUs in the lungs, whereas mice receiving INH plus Luteolin established a pathogen load of 2.12 X 10^5^ (5.3 Log) CFUs in the lung at 15 days following re-infection (Fig 6A and 6B). Thus, Luteolin can reduce TB relapses. Moreover, in the reactivation model, while 80% of the mice receiving INH treatment exhibited disease relapse, only 30% of the mice receiving INH plus Luteolin treatment relapsed (Fig 6C and 6D). These findings thus demonstrated that Luteolin-induced T_CM_ responses can effectively promote sterile immunity.

### Luteolin-induced protection can be adoptively transferred

**Fig 7:**
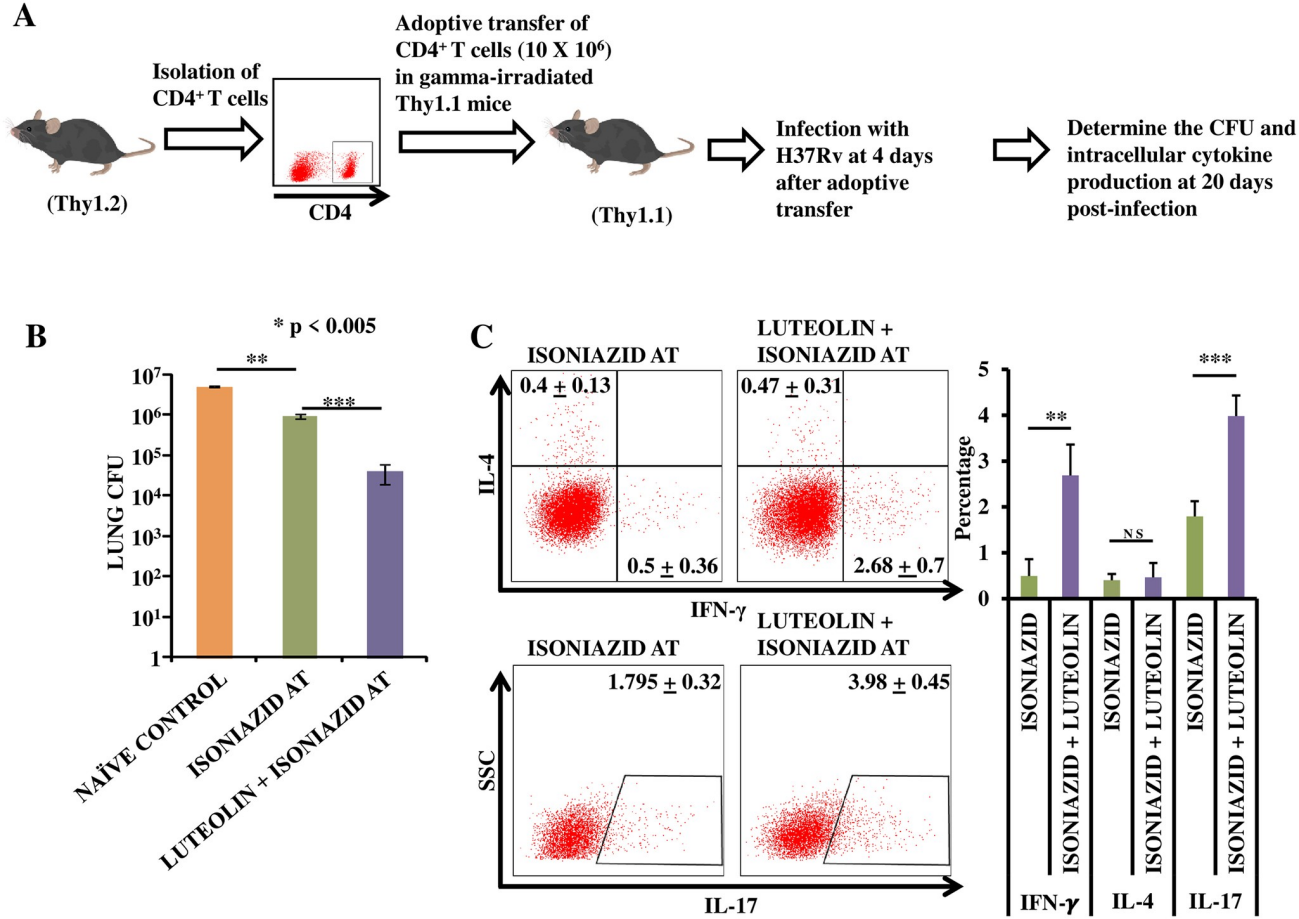
Adoptively transferred T cells from Luteolin-treated mice confer improved protection against TB. **(A)** Experimental Layout: CD4^+^ T cells were isolated from congenic wild-type Thy1.2^+^ mice infected with H37Rv followed by Luteolin treatment for 120 days and rested for 30 days. CD4^+^ T cells (10x10^6^) were adoptively transferred into γ-irradiated (sub-lethal dose of 800 rads/mice) Thy1.1^+^ congenic animals followed by infection with H37Rv. Twenty days after infection, spleen and lungs were isolated. **(B)** CFUs were estimated from lung homogenates of the different groups. **(C)** Splenocytes were challenged with H37Rv complete soluble antigen *ex vivo*. T cells were then stained for the intracellular cytokines IL-4 vs. IFN-γ, and IL-17. The results shown are representative of one experiment with six mice within each group, of which only 4 mice in the control group and 3 mice in the ISONIAZID AT group and 5 mice in the LUTEOLIN + ISONIAZID AT group survived by day 20. Differences were considered significant at P<0.05 and are represented by * p<0.05, ** p<0.01, *** p<0.001, **** p<0.0001 whereas non-significant differences are denoted by (NS).

In order to accurately assess the role of Luteolin-induced T_CM_ enrichment in protection against TB and to rule out an important contribution of the other immunostimulatory functions of Luteolin, we performed T cell adoptive transfer experiments. Congenic wild-type Thy1.2 mice were infected with *M*.*tb* H37Rv via the aerosol route and treated with INH alone or with INH plus Luteolin for 120 days and then rested for 30 days. CD4^+^ T cells (10x10^6^) were adoptively transferred into γ-irradiated (sub-lethal dose of 800 rads/mice) Thy1.1 congenic animals followed by infection with an aerosol challenge of *M*.*tb* H37Rv. Twenty days after infection, spleen and lungs isolated from the surviving mice in each group were evaluated for CFUs (Fig 7A and 7B) and antigen-specific intracellular cytokine responses (Fig 7A and 7C). Results showed that recipient mice receiving T cells from Luteolin-treated animals exhibited reduced CFUs (Fig 7B) and increased IFN-γ- and IL-17-producing, donor-derived (Thy1.2^+^) CD4^+^ T cells (Fig 7C).

### Luteolin alleviates Isoniazid-induced hepatotoxicity

Antitubercular therapy-induced hepatotoxicity often results in treatment cessation in patients, which further increases the risk for the generation of drug-resistant strains [4–6]. We therefore investigated effects of Luteolin on INH-mediated hepatotoxicity. Consistent with published studies, INH administration induced hepatotoxicity [10,48], as indicated by elevated serum levels of Alanine Aminotransferase (ALT), Aspartate Aminotransferase (AST) and Alkaline Phosphatase (ALP) (S3 Fig). Luteolin treatment alleviated INH-induced hepatotoxicity (S3 Fig). Thus, luteolin as an adjunct to DOTS may further improve treatment outcome by preventing DOTS-induced hepatotoxicity (and subsequent treatment cessation), thereby further preventing the generation of drug-resistance.

## Discussion

*M*.*tb* co-evolved with its human host and, therefore, armed itself with multiple host immune evasion mechanisms during the course of evolution. Only a small number of people (~10%) develop symptomatic disease immediately upon exposure to TB. The majority of exposed individuals successfully contain the *M*.*tb* organisms but, instead of completely eliminating them, develop latency [49,50]. The immune system of these individuals is only effective in confining the bacteria into restricted sites known as granulomas, and progression to active disease occurs only when the immune system is perturbed. Consequently, these pseudo-resistant individuals are susceptible to the development of active TB during HIV infection, treatment with corticosteroids or immunosuppressive drugs, alcohol abuse, etc [49,50]. Taken together, these findings indicate that the host immune system is sufficient to confine *M*.*tb*. Host-directed therapy involving immunomodulation therefore shows potential to enhance the efficacy of TB therapies. Studies with experimental animals and human subjects have shown that Th1 immune responses play a central role in host protective immunity against *M*.*tb* infection [13–15]. On the other hand, Th2 cells and Tregs facilitate disease progression by inhibiting host protective Th1 responses. Interestingly, Th17 cells play a crucial role in host resistance during secondary infections [51]. Recently, we have shown that simultaneous inhibition of Th2 cells and Tregs significantly reduces tubercular burden in infected animals but is unable to completely clear *M*.*tb* organisms [52]. This mode of treatment highly polarizes immune responses towards the Th1 phenotype, which causes severe inflammation and is detrimental to the host. Nevertheless, TB patients mount strong DTH responses, which are indicative of strong Th1 responses and, thus, Th1 responses may be critical but not sufficient for complete eradication of the harboured organisms. TB pathogenesis is contributed by two components: damage directly induced by the organisms, and damage caused by inflammation. In fact, addition of steroids during antibiotic treatment has been reported to be beneficial to patients [22]. Accordingly, addition of immunomodulators to antibiotics may represent a viable option for developing effective TB treatment regimens. It is now well-established that DOTS treatment causes hepatotoxicity, and a sizable number of patients withdraw from treatment because of this hepatotoxicity [48]. Furthermore, the recent literature suggests that current antibiotic therapy adopted by DOTS impairs host protective immune components, leaving treated patients particularly vulnerable to TB reactivation and reinfection [7–9]. We have recently shown that prolonged treatment with antitubercular antibiotics induces apoptosis in activated CD4^+^ T cells and this phenomenon renders animals hypersensitive to TB re-activation and re-infection [10]. Due to their critical role in host protection, Th1 and Th17 cells are vitally important for optimal vaccine efficacy against TB [51,52]. An immunomodulator that enhances and/or protects these Th cell subsets from cell death induced by antibiotics is highly desired. Prior studies have shown that Luteolin, in addition to hepatoprotective effects, is also efficacious against several infections and a variety of other diseases that are associated with TB susceptibility [29–31]. Furthermore, Luteolin has been shown to promote T_CM_ responses during the priming phase resulting in stronger T cell recall responses upon re-exposure [32]. While the immunomodulatory effects of Luteolin were studied in a vaccination model, we further interrogated the utility of Luteolin as adjunct to INH therapy as a potential host-directed therapy (HDT). Our results demonstrate that addition of Luteolin during TB therapy enhances T_CM_ responses resulting in potent Th1 and Th17 effector responses, leading to enhanced clearance of *M*.*tb* organisms from the host. A reduction in the treatment length is an important step for reducing the risk of developing drug-resistance and hence addition of Luteolin to current treatment regimens may prove beneficial for reducing the generation of drug-resistant *M*.*tb* variants.

Naïve T (CCR7^+^CD45RA^+^) and T_CM_ (CCR7^+^CD45RA^−^) cells require antigen priming in lymph nodes before they migrate to inflammatory sites, whereas terminally differentiated T_EM_ (CCR7^−^CD45RA^−^) cells rapidly enter inflamed tissues, produce copious amounts of IFN-γ and IL-4, and exhibit immediate effector function [53,54]. Terminally differentiated human CCR7^−^CD45RA^−^T_EM_ cells have been reported to strongly upregulate Kv1.3 K^+^ ion channel expression. Resting (unstimulated) T cells of all subsets express about 200–400 Kv1.3 channels per cell along with very few calcium-activated K^+^ (IKCa1) channels. After activation, CD4^+^ and CD8^+^ naïve T and T_CM_ cells upregulate IKCa1 (500–600 IKCa1 channels expressed per cell), whereas T_EM_ cells upregulate Kv1.3 (1,500–1,800 Kv1.3 channels expressed per cell) [55]. In fact, the Kv1.3^high^IKCa1^low^ phenotype has been suggested as a specific functional marker for activated T_EM_ cell subsets belonging to both the CD4^+^ and CD8^+^ compartments [55]. Since T_EM_ cells express significantly higher Kv1.3 levels and lower IKCa1 levels than naïve T and T_CM_ cells post-activation, selective Kv1.3 channel blockade suppresses proliferation of T_EM_ cells without affecting naïve T or T_CM_ cells [55]. Selective inhibition of Kv1.3 channels therefore appears to be a potential approach for generating T_CM_-driven long-term protective immunity against TB. While robust and rapid recall responses critically rely on the maintenance of memory T cells, this may also have detrimental effects such as depletion of the T_CM_ pool due to recurrent clonal expansion of T_EM_ cells during persistent infection by the same pathogen or due to recurrent exposure to related non-pathogenic strains [27,56]. The critical role of Kv1.3 ion channels in effector T cell function has already been explored as a target to suppress effector T cell functions in severe inflammatory diseases [57,58]. Kv1.3 loss of function in mice caused a delay in T_CM_ to T_EM_ differentiation by inhibiting cell cycle progression at the G2/M stage. This was mediated by up-regulation of SMAD3 phosphorylation, accelerating its translocation to the nucleus, where SMAD3 binds with the p21^cip1^ promoter and subsequently suppresses expression of the cell cycle genes cyclin-dependent kinase (Cdk)1 and cyclin B1 [36]. Nevertheless, Kv1.3-deficient mice were otherwise healthy and developed a normal immune system, with similar proportions of T lymphocytes in the spleen and thymus and similar proliferative responses of splenocytes to challenge with Concanavalin A or anti-CD3 antibodies when compared to wild type control mice [59]. A recent study reported that blockade of Kv1.3 by addition of clofazimine during immunization of mice with BCG enhances the available pool of T_CM_ cells, which provides superior protection against pulmonary TB [27]. Our study confirms these findings and further shows that Kv1.3 inhibition by Luteolin treatment effectively alters the T_CM_:T_EM_ cell ratios, and demonstrates improved protection against *M*.*tb* infection. Luteolin generated an expanded T_CM_ pool that dictated efficient recall responses, which are considered imperative for efficacy and longevity of protective immune responses (Fig 7). T_CM_ cells are believed to be a perpetual source of T_EM_ cells, which are responsible for protection from infections by induction of a rapid effector response. Earlier reported effects of Luteolin on NK cell function [38] were confirmed here in a primary infection model of TB, indicating that Luteolin-induced NK cells further enhance vaccine-induced protection against *M*.*tb*, likely in an IL-22-dependent manner [46].

In summary, we have shown that Luteolin treatment alters the T_CM_:T_EM_ cell ratio to induce improved immunity against TB. Additionally, Luteolin augments and reduces the duration of conventional anti-mycobacterial therapy, as it accelerates clearance of *M*.*tb* by inducing enhanced T_CM_ responses and augmenting Th1 and Th17 responses. Whether Luteolin can improve disease protection in individuals already immunized with BCG will be a topic for future investigation.

## Materials and methods

### Ethics statement

Animal experiments performed were in accordance with the guidelines approved by the 53rd Meeting of the Institutional Animals Ethics Committee held on 11^th^ February, 2014 at International Centre for Genetic Engineering and Biotechnology (ICGEB) (Approval ID: ICGEB/AH/2014/01/RGP-13), New Delhi, India as well as guidelines issued by the Department of Biotechnology (DBT), Government of India. All mice used for experiments were ethically sacrificed by asphyxiation in carbon dioxide according to institutional and DBT regulations.

### Mice

C57BL/6 mice that were Thy1.1^+^ or Thy1.2^+^ were initially purchased from The Jackson Laboratories (Bar Harbor, ME) and thereafter maintained at our specific pathogen-free animal facility at ICGEB. Mice used for infections were housed under barrier conditions in the Tuberculosis Aerosol Challenge Facility (TACF) of ICGEB and treated humanely as per the specified Animal Care protocols.

### *M*.*tb* aerosol infection of mice

*M*.*tb* strain H37Rv (ATCC 27294; American Type Culture Collection, Rockville, MD) was a kind gift from the Colorado State University repository (Fort Collins, CO). Mouse infections were performed in accordance to the aerosol infection model using a Madison Aerosol Chamber (University of Wisconsin, Madison, WI) with the nebulizer pre-calibrated at ~220 CFU/mice. *M*.*tb* strain H37Rv was grown to mid-log phase (OD_600_ ∼0.6) in Middlebrook 7H9 media (Difco, USA) with 0.1% Tween 80 (Sigma, USA), 0.2% glycerol and 10% Middlebrook albumin, dextrose and catalase (ADC) enrichment medium (Difco, USA). Bacteria were stored at −80°C in 20% glycerol stocks for further experiments. For aerosol infection, cultures were washed twice with PBS and made into a single cell suspension by passing through a 26-gauge syringe ten times following two passes through a 30-gauge syringe. Fifteen ml of the *M*.*tb* H37Rv single cell suspension (20X10^6^ cells per ml) was placed in the Nebulizer reservoir of the Madison Aerosol Chamber calibrated to deliver the desired CFUs of bacteria into the lungs of mice kept in the chamber in 15 minute cycles. At 24 hours after aerosol challenge 3 mice were euthanized for quantification of pathogen delivery to lungs by measuring CFUs in lung homogenates. Mice were found to be infected with ∼220 CFU of *M*.*tb* H37Rv in their lungs. The mice were maintained under BSL-3 containment thereafter. Treatment was started 15 days post infection and three randomly selected mice from each group were euthanized at different time points for CFU estimation or immunological studies.

### Drug administration

Five mg/kg of Luteolin (Sigma, USA) in 100 μl of PBS containing 5% DMSO v/v was administered intraperitoneally every day during the entire treatment phase, whereas controls were given vehicle only. INH (50 mg/L) was given *ad libitum* in the drinking water during therapy.

### Quantification of pathogen burden by Colony Forming Units (CFU)

Randomly selected mice were sacrificed at different time points, organs were harvested, homogenized in 0.2 μm filtered PBS containing 0.05% Tween 80 and plated onto 7H11 Middlebrook plates containing 10% OADC supplement. One hundred-fold, one thousand-fold, and ten thousand-fold diluted lung cell homogenate and ten-fold and one hundred-fold diluted spleen & liver cell homogenates were plated in doublet on 7H11 plates and incubated at 37°C for ~21 days. CFUs were counted and pathogen burden in lung, liver and spleen was estimated.

### Flow cytometry: Surface and intracellular staining

Spleens and lungs were isolated from mice and macerated by frosted slides in ice cold RPMI 1640 (Gibco, Invitrogen, UK) containing 10% FBS to prepare a single cell suspension. Red blood cells (RBCs) were lysed with RBC cell lysis buffer, incubated at room temperature for 3–5 minutes and washed with 10% RPMI 1640. The cells were counted and 1×10^6^ cells were used for surface staining. For intracellular staining 1×10^6^ cells were cultured per well in 12-well plates (Nunc, USA) and activated with 1μg/ml H37Rv Complete Soluble Antigen (CSA) overnight, and 10 μg/ml Brefeldin A (eBiosciences, USA) was added during the last 6 hours of culture. Cells were washed twice with PBS and stained with antibodies directed against surface markers. After staining, cells were washed again with PBS and fixed with 100 μl fixation buffer (eBiosciences, USA) for 30 minutes, then re-suspended in 200 μl permeabilization buffer (eBiosciences, USA) and stained with fluorescently labelled anti-cytokine antibodies. Fluorescence intensity of fluorochrome-labelled cells was measured by flow cytometry (FACS Canto II, BD Biosciences, USA). FACS Diva was used for acquiring the cells and final data analysis was performed by Flow Jo (Tree star, USA).

### Antigen-specific degranulation assay

Splenocytes isolated from randomly selected mice in different groups were isolated as described above and 2X10^6^ splenocytes per well were cultured in RPMI 1640 (Gibco, Invitrogen, UK) containing 10% FBS in a 12-well plate (Nunc, USA) at 37°C in 5% CO_2_ for 4 hours to bring the cellular activity to basal levels. Splenocytes were then challenged with 20 μg/ml of H37Rv CSA and cultured for an additional 2 hours, after which 5 μl Monensin (Golgi-Stop, BD Biosciences) and 5 μl of anti-CD107A-FITC antibody (BD Biosciences) were added per well and cultured for an additional 4 hours at 37°C in 5% CO_2_. These cells were then collected and surface-stained for FACS analysis.

### *M*.*tb* reactivation experiments

Mice infected with *M*.*tb* strain H37Rv following the aerosol infection model (∼220 CFU per mice) were treated with 50 mg/L INH administered *ad libitum* (in the drinking water) or 50 mg/L INH administered *ad libitum* along with 5 mg/kg Luteolin administered intraperitoneally every day for 120 days starting at day 16 after infection. These mice were then rested for 30 days followed by treatment with dexamethasone (5 mg/kg administered intraperitoneally) three times per week for 30 days. Ten mice from each group were then sacrificed and CFUs were estimated from lung homogenates to determine the reactivation rate of latent mycobacteria.

### *M*.*tb* reinfection experiments

To evaluate susceptibility to reinfection C57BL/6 mice were infected with *M*.*tb* strain H37Rv and treated with 50 mg/L INH administered *ad libitum*, either alone or with 5 mg/kg Luteolin administered intraperitoneally every day for 120 days starting at day 16 after infection. These mice were then rested for 30 days followed by reinfection with *M*.*tb* using the same dose and protocol as for the primary infection. Ten mice per group were then sacrificed at 15 days post-infection to evaluate the frequency of reinfection.

### T cell adoptive transfer experiments

For adoptive transfer experiments, Thy1.1^+^ mice were gamma-irradiated (4.6 rads/sec for 175 seconds) and rested for a day. CD4^+^ T cells were isolated using anti-CD4 beads over a magnetic column (MiltenyiBiotec, USA) from the lymph nodes of Thy1.2^+^ animals that were either naïve (Control) or had been previously infected with H37Rv and treated with either Isoniazid (ISONIAZID AT) or Luteolin in combination with Isoniazid (LUTEOLIN + ISONIAZID AT) for 120 days and then rested for an additional 10 days. These cells were then adoptively transferred into the irradiated recipient mice (10x10^6^ cells per mouse). After 5 days recipient mice were challenged with *M*.*tb* H37Rv through the aerosol route. Each group was comprised of 6 mice, of which, 4 mice in the control group and 3 mice in the ISONIAZID AT group and 5 mice in the LUTEOLIN + ISONIAZID AT group survived on day 20 post-infection. The surviving mice were euthanized for CFU estimation or immunological studies.

### Hepatotoxicity assays

Serum activity of Alanine Aminotransferase (ALT), Aspartate Aminotransferase (AST) and Alkaline Phosphatase (ALP) were used as indicators of host hepatotoxicity. These assays were performed by using diagnostic kits obtained from Span Diagnostic Limited (India), in accordance with the manufacturer’s protocol. Sera from 6 randomly chosen mice from each group were used.

### Antibodies and reagents

We used the following antibodies: anti-CD3 (clone: 145-2C11)-PerCP-Cy5 or -APC, CD4 (clone: GK1.5, RM4-5)-FITC, -PerCP-Cy5 or -APC, CD8 (clone: 53–6.7)-FITC, -APC-H7, -PerCP-Cy5 or -APC, -NK1.1 (clone: PK136)-Alexa 700, -PerCP-Cy5 or -PE, CD44 (clone: IM7)-APC, CD62L (clone: MEL-14)-PE, CD25 (clone: 3C7)-PE, -APC, FOXP3 (clone: MF23, R16-715)-APC, IFN-γ (clone: XMG1.2)-APC, IL-4 (clone: 11B11)-PE, IL-6 (clone: MPS-20F3)-PE, IL-10 (clone: JES5-16E3)-APC, IL-12 (clone: C15.6)-PE, IL-17 (clone: O79-289)-PE, IL-2 (clone: JES6-5H4)-PerCP or -FITC, IL-22 (clone: Poly5164)-PE, TNF-α (clone: MP6-XT22)-PE, CD107A (clone: 1DB4)-FITC (all from BD Biosciences, USA), TGF-β (clone: TW7-16B4)-APC (from Biolegend, USA), and CD69 (clone: H1.2F3)-PE (from eBiosciences, USA).

### qRT-PCR analysis

Single cell suspension was prepared from the lungs of infected mice by maceration using frosted slides in complete RPMI media followed by Red blood cells (RBCs) lysis. Total RNA was isolated from the lung cells using RNAeasy isolation kit (QIAGEN, Germany) according to the manufacturer’s protocol. RNA isolation was followed by cDNA preparation using iscript cDNA synthesis kit (Bio-Rad). qRT-PCR was performed using SYBR Green Master Mix (Bio-Rad) on BioRad Real-Time thermal cycler (BioRad, USA). *ACTB* was used as an internal control. The primers used in this study are listed in Table 1.

**Table 1:** Primers used for qRT-PCR analysis.

	Forward Primer (5’-3’)	Reverse primer (5’-3’)
***IFNγ***	ATGAACGCTACACACTGCATC	CCATCCTTTTGCCAGTTCCTC
***IL4***	GGTCTCAACCCCCAGCTAGT	GCCGATGATCTCTCTCAAGTGAT
***IL17***	TTTAACTCCCTTGGCGCAAAA	CTTTCCCTCCGCATTGACAC
***IL12***	CTGTGCCTTGGTAGCATCTATG	GCAGAGTCTCGCCATTATGATTC
***IL6***	TAGTCCTTCCTACCCCAATTTCC	TTGGTCCTTAGCCACTCCTTC
***TGFβ***	CTCCCGTGGCTTCTAGTGC	GCCTTAGTTTGGACAGGATCTG
***IL22***	ATGAGTTTTTCCCTTATGGGGAC	GCTGGAAGTTGGACACCTCAA
***TNFα***	CCCTCACACTCAGATCATCTTCT	GCTACGACGTGGGCTACAG
***IL10***	GCTCTTACTGACTGGCATGAG	CGCAGCTCTAGGAGCATGTG
***IL2***	TGAGCAGGATGGAGAATTACAGG	GTCCAAGTTCATCTTCTAGGCAC
***ACTB***	AGTGTGACGTTGACATCCGTAAAGA	GGACAGTGAGGCCAGGATGG

### Statistical analysis

All data were derived from at least two independent experiments unless specified otherwise. Statistical analyses were conducted using Graphpad Prism v8 software and values were presented as mean with standard deviations. Significant differences between the group means were determined by one way analysis of variance (ANOVA) followed by *Post-hoc* analysis with Tukey’s correction for Multiple comparison. Differences were considered significant at P<0.05. For comparing the relapse experiment depicted in Fig 6A Mann-Whitney U test and Fig 6C Chi-square test was used.

## Supporting information

S1 Fig *In vitro M*.*tb* growth curve Assay to assess the antimycobacterial activity of Luteolin. **(A)** OD of the H37Rv in the presence of Luteolin and Isoniazid. **(B)** Statistical significance of the growth curve. **(C)** CFU of the H37Rv in the presence of Luteolin and Isoniazid.
(TIF)

S2 Fig (A)Gating strategy and (B) Isotype controls for flow cytometry panels. (TIF)

S3 Fig Luteolin treatment alleviates Isoniazid-induced hepatotoxicity. Serum isolated from randomly chosen mice of different study groups 60 days post treatment were processed for estimation of **(A)** serum ALT activity, **(B)** serum AST activity, and **(C)** serum ALP activity.
(TIF)
