# Supplementary figures and images for: Luteolin as a potential host-directed immunotherapy adjunct to isoniazid treatment of tuberculosis
Source: PLoS Pathog. 2021 Aug 20;17(8):e1009805. doi: 10.1371/journal.ppat.1009805 (PMC8409628; doi:10.1371/journal.ppat.1009805)

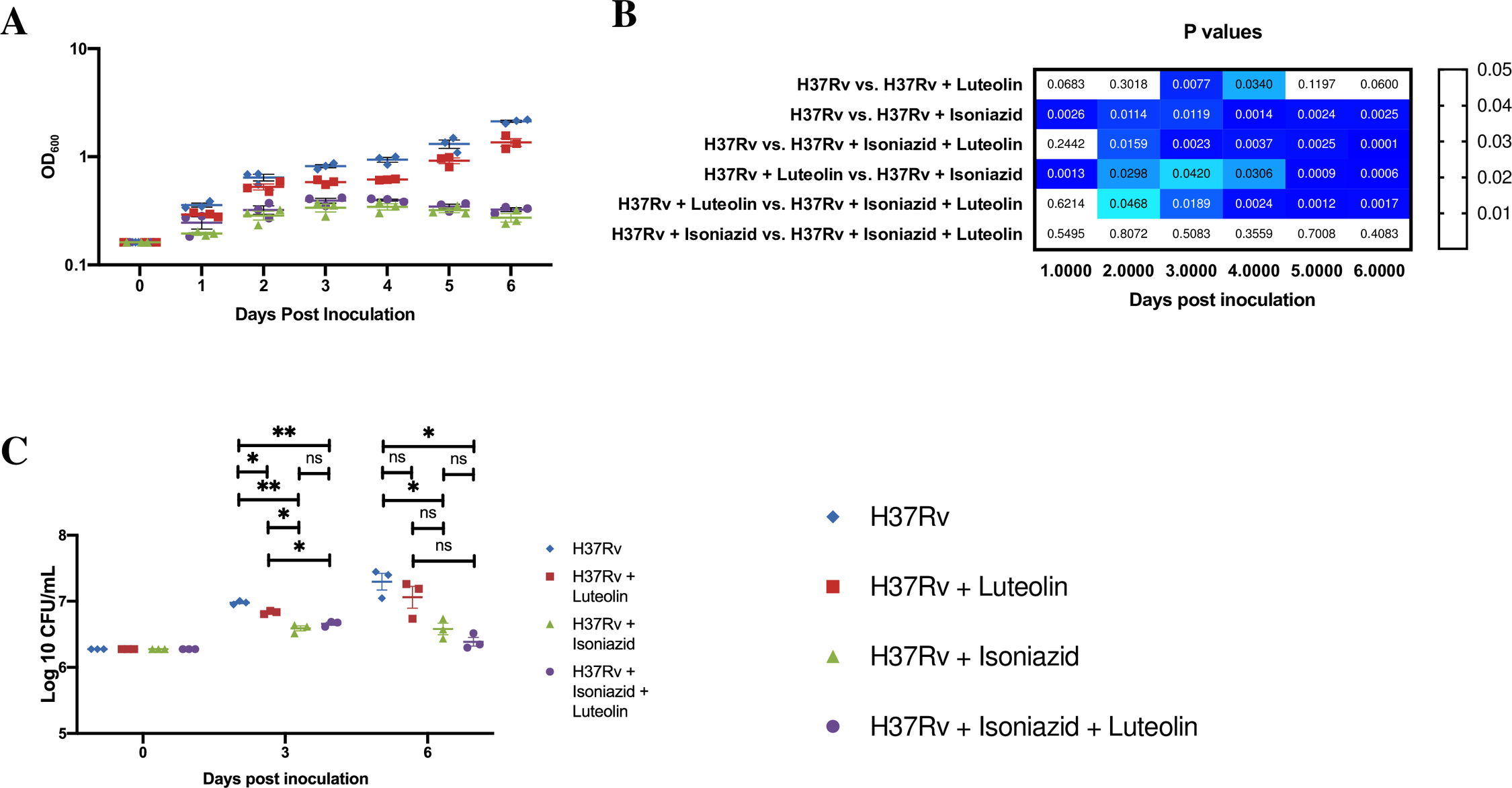

Supplement: S1 Fig — (A) OD of the H37Rv in the presence of Luteolin and Isoniazid. (B) Statistical significance of the growth curve. (C) CFU of the H37Rv in the presence of Luteolin and Isoniazid. (TIF) [file ppat.1009805.s001.tif]

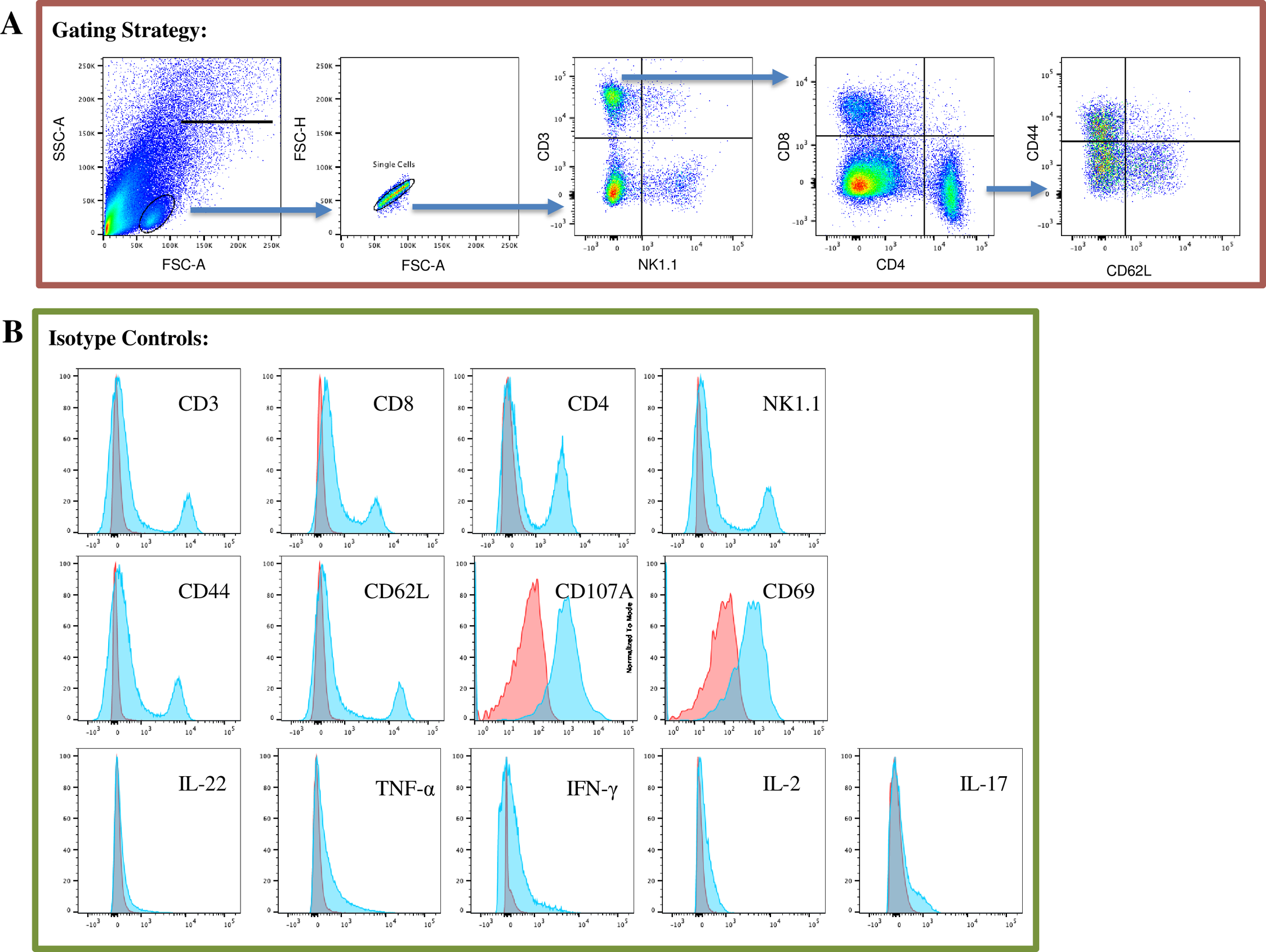

Supplement: S2 Fig — (TIF) [file ppat.1009805.s002.tif]

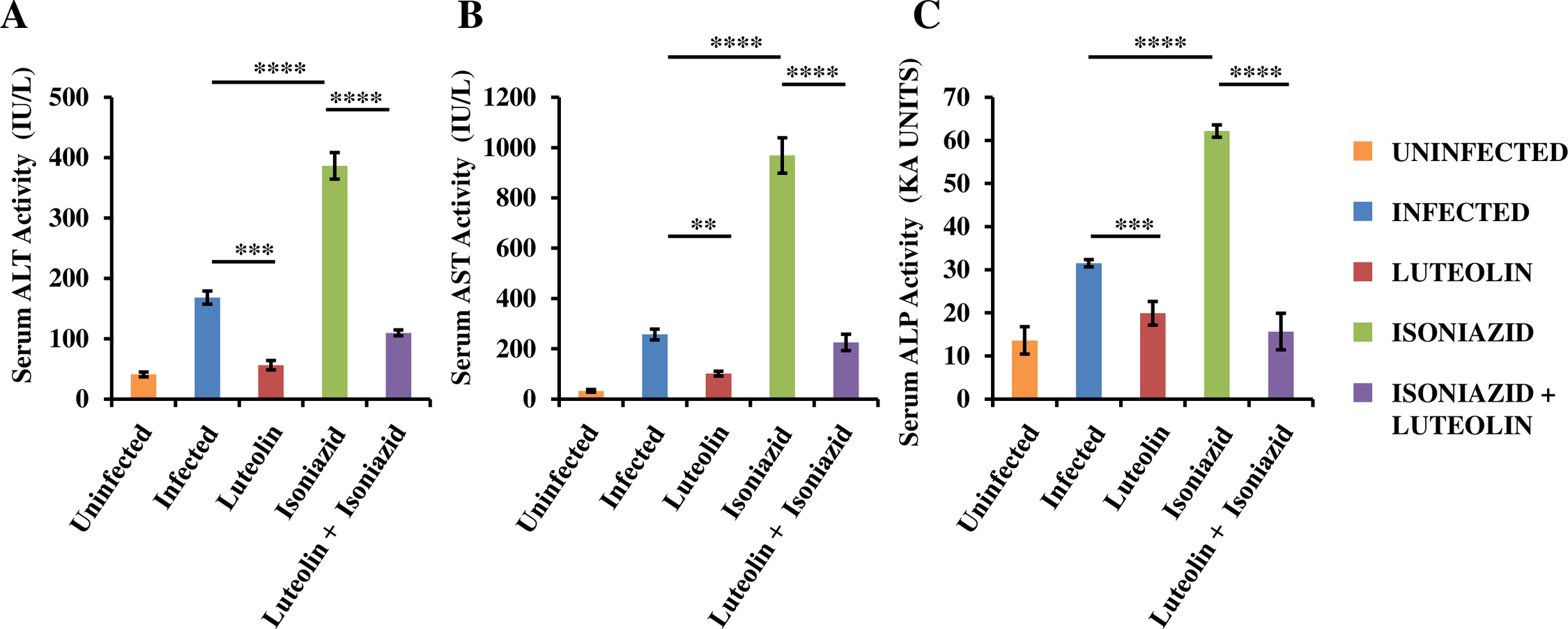

Supplement: S3 Fig — Serum isolated from randomly chosen mice of different study groups 60 days post treatment were processed for estimation of (A) serum ALT activity, (B) serum AST activity, and (C) serum ALP activity. (TIF) [file ppat.1009805.s003.tif]
